# Contribution of traditional deep fermentation to volatile metabolites and odor characteristics of Wuyi rock tea

**DOI:** 10.3389/fbioe.2023.1193095

**Published:** 2023-05-16

**Authors:** Xiaoli Jia, Yuhua Wang, Qisong Li, Qi Zhang, Ying Zhang, Shaoxiong Lin, Pengyuan Cheng, Meihui Chen, Mengru Du, Jianghua Ye, Haibin Wang

**Affiliations:** ^1^ College of Tea and Food, Wuyi University, Wuyishan, China; ^2^ College of Life Science, Fujian Agriculture and Forestry University, Fuzhou, China; ^3^ College of Life Science, Longyan University, Longyan, China

**Keywords:** Wuyi rock tea, fermentation, volatile metabolome, odor characteristics, characteristic compound

## Abstract

Fermentation is extremely important for the formation of the special flavor of Wuyi rock tea. This study determined volatile metabolite contents using GC-MS technique and futher analyzed their odor characteristics during the traditional deep fermentation technology of Wuyi rock tea. The results showed that 17 characteristic compounds significantly changed during the first stage of the preliminary processing, namely fresh leaves, withering and fermentation. The key to the formation of floral aroma lied in dihydromyrcenol, and the woody aroma derived from six terpenoids, and their synthesis depended on dihydromyrcenol content. The fruity aroma was dominated by six esters, and the fruity aroma mainly came from (Z) -3-hexen-1-yl butyrate, (E) -3-hexen-1-yl butyrate and 5-Hexenyl butyrate. This study provided an important theoretical and practical basis for improving the preliminary processing of Wuyi rock tea.

## 1 Introduction

Wuyi Mountain in Fujian Province, China is located between latitude 27°32′36 "∼ 27°55′15″ north and longitude 117°24′12 "∼ 118°02′50″ east. It is a World Cultural and Natural Heritage Site, a World Biosphere Reserve, one of China’ first national parks, as well as an important tea producing area in China and the birthplace of oolong tea. The geology of Wuyi Mountain is a typical Danxia landform, and the special geographical structure gave birth to a special tea - Wuyi rock tea. Wuyi rock tea is a typical oolong tea, and is semi-fermented and its production process is more complex than other types of tea (Wong et al., 2022; Ye et al., 2023). High-quality Wuyi rock tea should have distinct characteristics such as fruity, floral, woody and fatty, and the key to the production of these odor characteristics lied in the preliminary processing (Pang et al., 2022; Xu et al., 2022). The preliminary processing of Wuyi rock tea was mainly divided into two stages. The first stage was fresh leaves picking, withering and fermentation, and in this stage, tea leaves underwent simultaneous enzymatic and nonenzymatic reactions after picking fresh leaves, and the transformation between compounds was the most intense at this stage, which was also the key link to forming the odor characteristics of Wuyi rock tea (Liu et al., 2022a; Wu et al., 2022). The second stage consisted of green removing, kneading and drying, and was mainly the transformation the substance generated by the nonenzymatic reaction, the basis of which was derived from the first stage of preliminary processing (Wang et al., 2022a; Yang et al., 2023). Therefore, the first stage of processing was extremely important in the formation of the odor characteristics of Wuyi rock tea during the preliminary processing.

The first stage of the preliminary processing of Wuyi rock tea mainly consisted of three steps, namely tea fresh leaf picking, tea withering and tea fermentation. The picking of Wuyi rock tea still followed the traditional picking method, mainly picking 3–4 leaves of tea tree bud. After picking, the tea leaves began to wither, when the fresh tea leaves began to lose water and soften, and the substances in the tea leaves began to transform and accumulate, laying the foundation for subsequent processing (Wang et al., 2022b). After withering, the tea leaves entered fermentation, which was an important link to the formation of key odor characteristics of Wuyi rock tea (Liu et al., 2023). The fermentation process of Wuyi rock tea was special and complex, and the fermentation of high-quality Wuyi rock tea required 5 to 6 operations, and during each fermentation process, the transformation of substances in the tea leaves constantly intensified, which finally resulted in the formation of a special flavor of Wuyi rock tea (Wang et al., 2020; Cai et al., 2022). During the fermentation process, the enzyme activity in tea leaves increased dramatically, and the change in enzyme activity promoted the transformation of substances in tea leaves, which was conducive to the accumulation of inclusions in tea leaves (Zhao et al., 2022). Secondly, fermentation time affected the degree of transformation of substances in tea leaves, and the appropriate time was more conducive to the formation of special flavor in tea leaves (Salman et al., 2022). The degree of fermentation determined the efficiency of substance transformation in tea leaves, and it was very important for the formation of different flavors in tea (Xiang et al., 2022; Zeng et al., 2022). It can be seen that the fermentation process was extremely important for the formation of odor characteristics and special flavor of tea leaves. The fermentation of Wuyi rock tea was extremely complicated, however, a large number of studies on the influence of processing on the formation of special flavor of Wuyi rock tea mainly focused on the processing process, and the influence of fermentation on the odor characteristics of Wuyi rock tea was only a simple analysis of fermentation results (Zhang et al., 2019; Chen et al., 2020; Guo et al., 2021), while in fact, the fermentation of Wuyi rock tea was a process of multiple fermentation to different degrees. Therefore, an in-depth analysis of the changes in volatile metabolites of tea leaves during the actual fermentation of Wuyi rock tea was of great significance to reveal the fermentation mechanism on the formation of special flavor of Wuyi rock tea and the production of high-quality Wuyi rock tea.

Accordingly, in this study, the tea leaves of Wuyi Rougui were processed according to the traditional Wuyi rock tea production processing process and collected fresh leaves, withered leaves and tea leaves with different fermentation processes to measure the volatile metabolites and analyze the effects of fermentation on volatile metabolites and odor characteristics of Wuyi rock tea, with a view to providing some reference for the processing of high-quality Wuyi rock tea.

## 2 Materials and methods

### 2.1 Field experiment and sample collection

Wuyi Mountain in Fujian Province, China belongs to the subtropical region, located at latitude 27°32′36"∼ 27°55′15″ north and longitude 117°24′12"∼ 118°02′50″ east, with an annual average temperature of 12–13°C, the annual precipitation more than 2000 mm, the relative humidity 85% and fog days more than 100 days.

**FIGURE 1:**
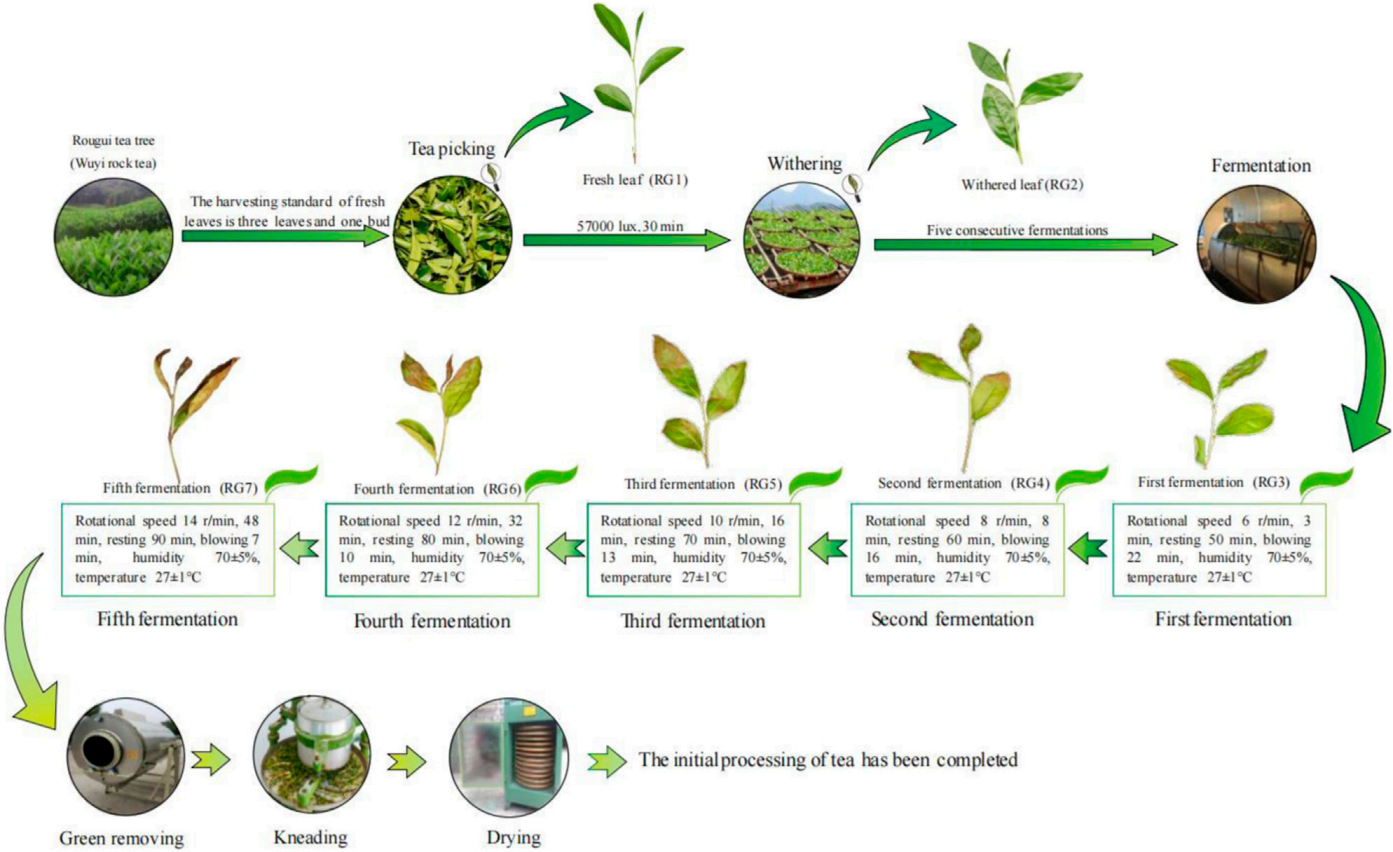
Traditional processing flow chart and sampling points of Wuyi rock tea. Note: RG1: Fresh leaf; RG2: Withered leaf; RG3: First fermentation; RG4: Second fermentation; RG5: Third fermentation; RG6: Fourth fermentation; RG7: Fifth fermentation; In this study, the tea leaves from RG1 to RG7 were collected for the determination of volatile metabolites.

Rougui tea tree is one of the main tea varieties planted in Wuyi Mountain, and in 1985 was identified as the provincial superior seed by Fujian Provincial Crop Variety Certification Committee, with a reputation of “no more fragrant than Rougui tea”. In May 2022, 600 kg of fresh leaves (three leaves and one bud) were collected from Rougui tea tree with 3 replicates of 200 kg each. Tea leaves were carried out preliminary processing according to traditional Wuyi rock tea production methods. The preliminary processing consisted of six steps: fresh leaves, withering, fermentation (five times continuous fermentation), green removing, kneading and drying (Wang et al., 2020). This study focused on the effect of fermentation and previous processing on volatile metabolites of Wuyi rock tea. Therefore, in this study, fresh tea leaves (RG1), withered tea leaves (RG2) and tea leaves after the 1st to 5th fermentation (RG3∼RG7) were collected, with 1 kg samples taken each time and 3 replicates. The volatile compounds of tea leaves were extracted, enriched and determined by gas chromatography-mass spectrometry (GC-MS) for volatile metabolites of tea (Figure1), with 3 replicates for each sample.

Therefore, fresh tea (RG1), wilted tea (RG2), and fermented tea 1–5 times (RG3∼(RG7)) were collected in this study with 1 kg samples per copy for 3 replicates. The collected tea leaves were extracted, enriched, and the volatile metabolites were determined by gas chromatography-mass spectrometry (GC-MS) (Figure 1).

### 2.2 Sample preparation and treatment

Tea leaves at different processing steps were collected, weighted, immediately frozen in liquid nitrogen, and stored at −80°C until needed. Samples were ground to powder using liquid nitrogen. 500 mg of the powder was immediately transferred to a 20 mL headspace vial (Agilent, Palo Alto, CA, USA), containing a saturated solution of NaCl, to inhibit any enzymatic reaction. The vials were sealed using crimp-top caps with TFE-silicone headspace septa (Agilent). For SPME analysis, each vial was placed in 60°C for 5 min and then 120 µm DVB/CWR/PDMS fibre (Agilent) was exposed to the headspace of samples at 100°C for 15 min.

### 2.3 GC-MS conditions

After sampling, volatile organic compounds (VOCs) in the fibre coating were desorbed in the injection port of the GC apparatus (Model 8,890; Agilent) in the splitless mode at 250°C for 5 min. The identification and quantification of VOCs were performed using an Agilent Model 8890 GC and a 7000D mass spectrometer (Agilent), equipped with a 30 m × 0.25 mm × 0.25 μm DB-5MS (5% phenyl-polymethylsiloxane) capillary column. Helium was used as the carrier gas at a linear velocity of 1.2 mL/min. The injector temperature maintaind at 250°C and the detector temperature at 280°C. The oven temperature was started from 40°C for 3.5 min, and increased at 10°C/min to 100°C, at 7°C/min to 180°C, at 25°C/min to 280°C, held for 5 min. Mass spectra were recorded in electron impact (EI) ionization mode at 70 eV. The temperatures of the quadrupole mass detector, ion source and transfer line were set at 150, 230°C and 280°C, respectively. MS with ion monitoring (SIM) mode was used for the identification and quantification of analytes.

### 2.4 Statistical analysis

Excel 2017 software was used to calculate the mean value and variance of the data, Rstudio 3.3 software was used to make box charts, principal component analysis (PCA), simulated trend chart, orthogonal partial least squares-discriminant analysis (OPLS-DA), volcanic map, bubble diagram, rose diagram and TOPSIS analysis; Heml 1.0 software was used to make heat maps.

## 3 Results and discussion

### 3.1 Analysis of volatile metabolites in tea leaves

**FIGURE 2:**
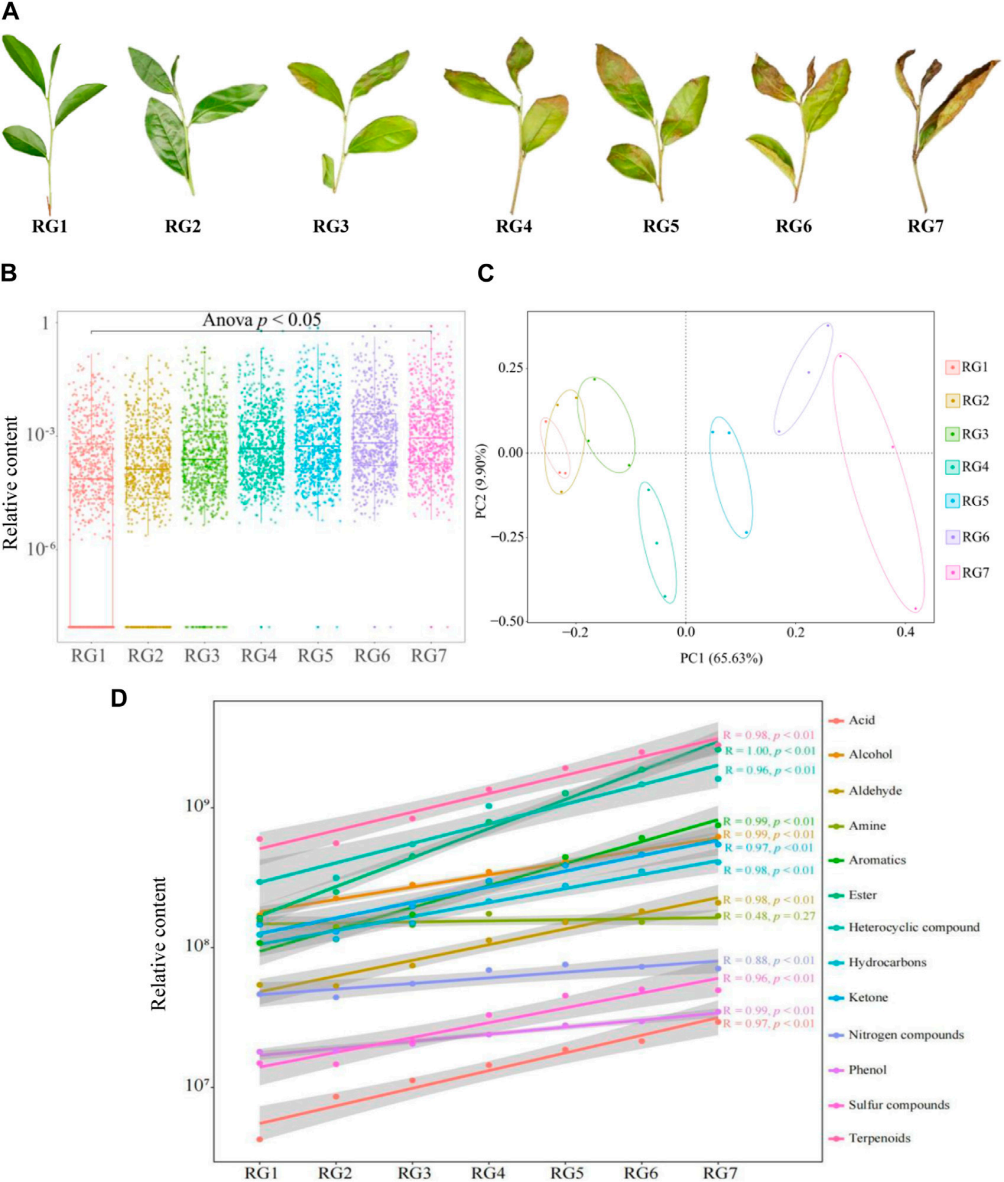
Metabolomic analysis of volatile compounds in tea leaves at different processing steps. Note: RG1: Fresh leaf; RG2: Withered leaf; RG3: First fermentation; RG4: Second fermentation; RG5: Third fermentation; RG6: Fourth fermentation; RG7: Fifth fermentation; **(A)** Morphological changes of tea leaves at different processing steps; **(B)** Analysis of the content of volatile metabolites in tea leaves at different processing steps; **(C)** Principal component analysis of the content of volatile metabolites in tea leaves at different processing steps. **(D)** The content analysis of different categories compounds in tea leaves with the extension of processing process.

The transformation of the substance during tea processing was mainly divided into two stages. Fresh leaves–withering–fermentation stage was the key link of tea processing, in which tea leaves underwent rapid transformation of substances under the simultaneous action of enzymatic and nonenzymatic reactions, while at the green removing–kneading–drying stage, tea leaves substance transformation was mainly through non-enzymatic reaction (Cheng et al., 2020; Li et al., 2022; Zhai et al., 2022). Tea fermentation was a complex process, but fermentation was conducive to the transformation of compounds in tea leaves under enzymatic and nonenzymatic reactions, and only through fermentation could the unique aroma and quality of tea be formed (Wang et al., 2020; Cao et al., 2021; Guo et al., 2022). The key to this study was to explore the changes of volatile metabolites in tea during the fresh leaf-withering-fermentation process. From Figure 2A, it can be seen that the morphology of tea leaves changed significantly from fresh leaves to withered leaves and then 5 fermented leaves. Therefore, volatile metabolites in tea leaves during the fresh leaves–withering–fermentation stage were measured by GC-MS technique in this study, and the results showed (Supplementary Table S1) that the types and amounts of volatile compounds detected in tea leaves at different steps were the same, and 754 volatile compounds were detected, among which the amounts of terpenoids, heterocyclic compounds, esters were the most, as shown 152 (20.16%), 124 (16.45%) and 120 (15.92%), respectively. Further analysis revealed (Figure 2B) that the content of volatile compounds in tea leaves increased significantly at different processing steps. Secondly, the analysis revealed (Supplementary Table S2) that the content of volatile compounds in tea leaves increased significantly from the first fermentation (RG3), and the top three categories of compounds were terpenoids, heterocyclic compounds and esters, and the content of the three categories of compounds showed a significant upward trend. The results of the analysis of the content percentage of different categories of compounds in tea leaves showed (Supplementary Table S3) that terpenoids, heterocyclic compounds and esters were all the highest at different steps. It has been reported that terpenoids, heterocyclic compounds, and esters compounds were closely related to tea aroma formation and were the main components of tea volatile compounds, and the higher their content, the stronger the tea aroma (Yun et al., 2021; Zhu et al., 2021; Li et al., 2023a). Therefore, the effect of fermentation on tea aroma might be related to changes in the content of terpenoids, heterocyclic compounds, and esters.

Principal component analysis was conducted for different processing steps of tea leaves based on volatile compound content, and the results showed (Figure 2C) that the two principal components could effectively distinguish the different processing steps of tea leaves, in which the contribution rate of principal component 1 was 65.63% and that of principal component 2 was 9.90%, with an overall contribution rate of 75.53%. Secondly, it can be seen from Figure 2C that fresh tea leaves (RG1) and withered tea leaves (RG2) were relatively similar in terms of volatile compound content, while the volatile compound content in tea leaves changed significantly from the first fermentation to the fifth fermentation (RG3∼RG5). Further analysis revealed (Figure 2D) that with the extension of tea processing, the content of different types of volatile compounds in tea leaves showed a significant upward trend, except for amine compounds. Liu et al. (2022b) found that the content of volatile compounds in Wuyi rock tea did not change much during the withering process, but the internal cell structure of tea leaves had changed significantly, which was conducive to the rapid transformation of compounds in subsequent fermentation. It can be seen that there was no significant difference in the quantity of volatile compounds in tea leaves during the tea fermentation process, while the content changed significantly. After withering, volatile compounds in tea leaves began to accumulate, and the content of volatile compounds increased significantly after the first fermentation and continued to rise with the increase in fermentation times. In addition, the most significant effects of fermentation on different categories of volatile compounds in tea leaves were terpenoids, heterocyclic compounds, and esters.

### 3.2 Analysis of the content of volatile compounds in tea leaves

**FIGURE 3:**
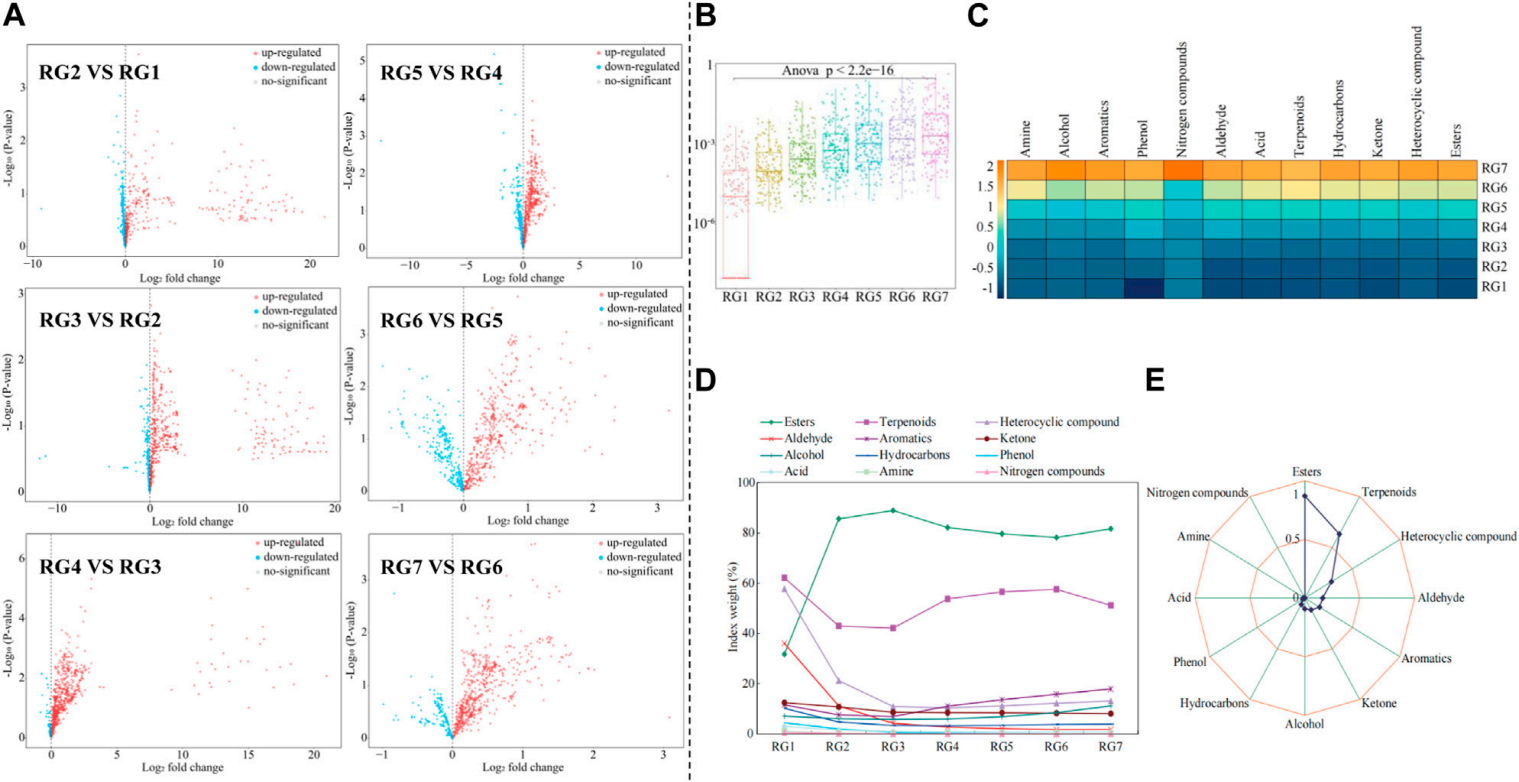
Analysis of the effect of different processing steps on volatile compounds in tea leaves and the index weight of different categories of compounds. Note: RG1: Fresh leaf; RG2: Withered leaf; RG3: First fermentation; RG4: Second fermentation; RG5: Third fermentation; RG6: Fourth fermentation; RG7: Fifth fermentation; **(A)** Volcanic map analysis of volatile compounds in tea leaves at different processing steps; **(B)** Analysis of the content of 179 volatile compounds at different processing steps; **(C)** Content analysis of 179 volatile compounds after classification; **(D)** TOPSIS index weight analysis of different processing steps on different categories of compounds; **(E)** TOPSIS index weight analysis of the comprehensive effect of processing steps on different categories of compounds.

In this study, we analyzed in depth the changes in volatile compound content in tea leaves during the fresh leaves–withering–fermentation stage, and the results showed (Figure 3A, Supplementary Table S4) that there were 389 upregulated and 249 downregulated volatile compounds after withering (RG2) compared to fresh tea leaves (RG1); 533 upregulated and 192 downregulated volatile compounds in tea leaves after the first fermentation (RG3) compared to RG2; 697 upregulated and 56 downregulated volatile compounds in tea leaves after the second fermentation (RG4) compared to RG3; 519 upregulated and 235 downregulated volatile compounds after the third fermentation (RG5) compared to RG4; 492 upregulated and 261 downregulated volatile compounds after the fourth fermentation (RG6) compared to RG5; 603 upregulated and 150 downregulated volatile compounds after the fifth fermentation (RG7) compared to RG6. It can be seen that the content of volatile compounds in tea leaves was constantly changing during processing. Secondly, the analysis revealed (Figure 3B) that, with the extension of the processing process (RG1∼RG7), a total of 179 volatile compounds in tea leaves showed a constantly increasing trend in content, while no compounds showed a constantly decreasing trend. Further classification analysis of 179 volatile compounds revealed (Supplementary Table S5) that they could be classified into 12 categories, of which terpenoids, heterocyclic compounds and esters compounds were the most, with 45 (24.14%), 31 (17.32%) and 31 (17.32%), respectively. The results of compound content analysis showed (Figure 3C, Supplementary Table S6) that with the extension of the processing process (RG1∼RG7), the content of 179 volatile compounds in tea leaves showed a significant increasing trend, among which, in the pre-fermentation stage of tea leaves (RG1∼RG3), esters, terpenoids and heterocyclic compounds were the most in the early stage of tea fermentation (RG1∼RG3), and esters, terpenoids and aromatics were the most in late tea fermentation (RG4∼RG7). Esters, terpenoids, heterocyclic compounds and aromatic compounds in tea leaves were important compounds in tea aroma formation, but the most dominant were terpenoids and esters, which had the highest content and proportion (Wang et al., 2022a; Luo et al., 2022). Terpenoids and esters as the main compounds constituting the aroma of tea were often collected and then returned to the tea to enhance the aroma of tea (Dalpathadu et al., 2022). It can be seen that during the fresh leaves–withering–fermentation stage, the content of different categories of volatile compounds in tea leaves changed significantly, but the most significant changes in the content of esters and terpenoids, which were extremely important for tea aroma formation, were always the highest.

On this basis, TOPSIS was further used in this study to analyze the contribution weights of different categories of volatile compounds in tea leaves at different steps. The results showed (Figure 3D) that the highest contribution weights of different categories of compounds in fresh tea leaves (RG1) were terpenoids, followed by heterocyclic compounds, aldehyde, and esters. When the tea leaves entered fermentation, the contribution weights of different categories of compounds changed significantly from RG2 to RG7, among which the contribution weights of esters and terpenoids remained the highest, especially esters. Therefore, the TOPSIS method was used again in this study to comprehensively evaluate the contribution weights of withering fermentation to different categories of volatile compounds in tea leaves, and the results showed (Figure 3E) that esters and terpenoids contained the highest contribution weights, and were mainly affected during the fresh leaves–withering–fermentation stage.

### 3.3 Screening of key compounds

**FIGURE 4:**
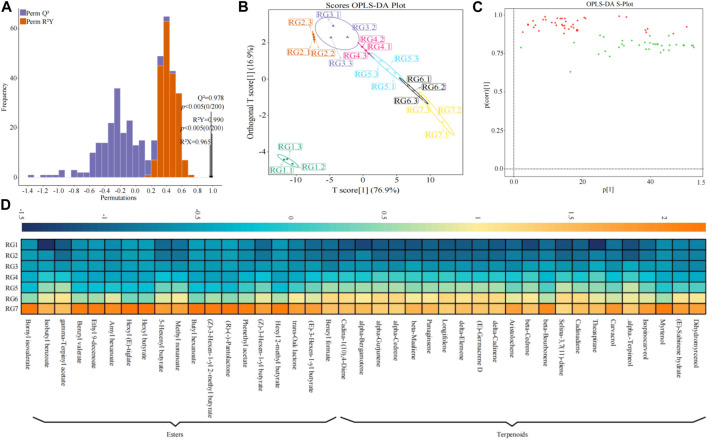
Screening of characteristic volatile compounds and analysis of odor characteristics of tea leaves at different processing steps. Note: RG1: Fresh leaf; RG2: Withered leaf; RG3: First fermentation; RG4: Second fermentation; RG5: Third fermentation; RG6: Fourth fermentation; RG7: Fifth fermentation; **(A)** The fitting test of OPLS-DA model; **(B)** OPLS-DA model scores of tea leaves at different processing steps; **(C)** Load diagram of OPLS-DA model; **(D)** Heat map analysis of key compounds (VIP >1) screened by OPLS-DA model.

OPLS-DA can be used to establish a correlation model between sample class and their compound content, and to screen markers that can characterize sample differences by variable importance projection value (VIP value) (Li et al., 2023b). Meanwhile, to test the reliability of the OPLS-DA model, permutation test was usually used to verify the model, so as to evaluate the accuracy of the model (Rivera-Pérez et al., 2022). Based on the above analysis, it was found in this study that the content of 179 volatile compounds constantly increased during tea processing, and tea processing mainly affected esters and terpenoids. In order to obtain the key volatile compounds in tea leaves at different processing steps, the OPLS-DA model was used to analyze the volatile compounds of esters and terpenoids. The results showed (Figure 4A) that the R2Y value of the goodness-of-fit of OPLS-DA model at different processing steps was 0.990 (*p* < 0.005), and the Q^2^ value of the prediction was 0.978 (*p* < 0.005). It can be seen that both R^2^Y and Q^2^ values of the model reached significant levels, indicating that the model had a good fitting degree and high reliability, which could be used for further analysis. The results of the OPLS-DA scoring chart showed (Figure 4B) that OPLS-DA could effectively distinguish samples at different processing steps into different regions of the coordinate chart, which showed significant differences in compound content at different processing steps. The results of the OPLS-DA loading diagram analysis showed (Figure 4C; Figure 4D) that 41 key compounds (VIP >1) could distinguish samples at different processing steps, including 22 terpenoids and 19 ester compounds, and the content of 41 compounds increased with the extension of the processing process. It can be seen that there were significant differences in the content of key volatile compounds in tea leaves at different processing steps.

### 3.4 Screening of characteristic compounds in key compounds and analysis of their odor characteristics

**FIGURE 5:**
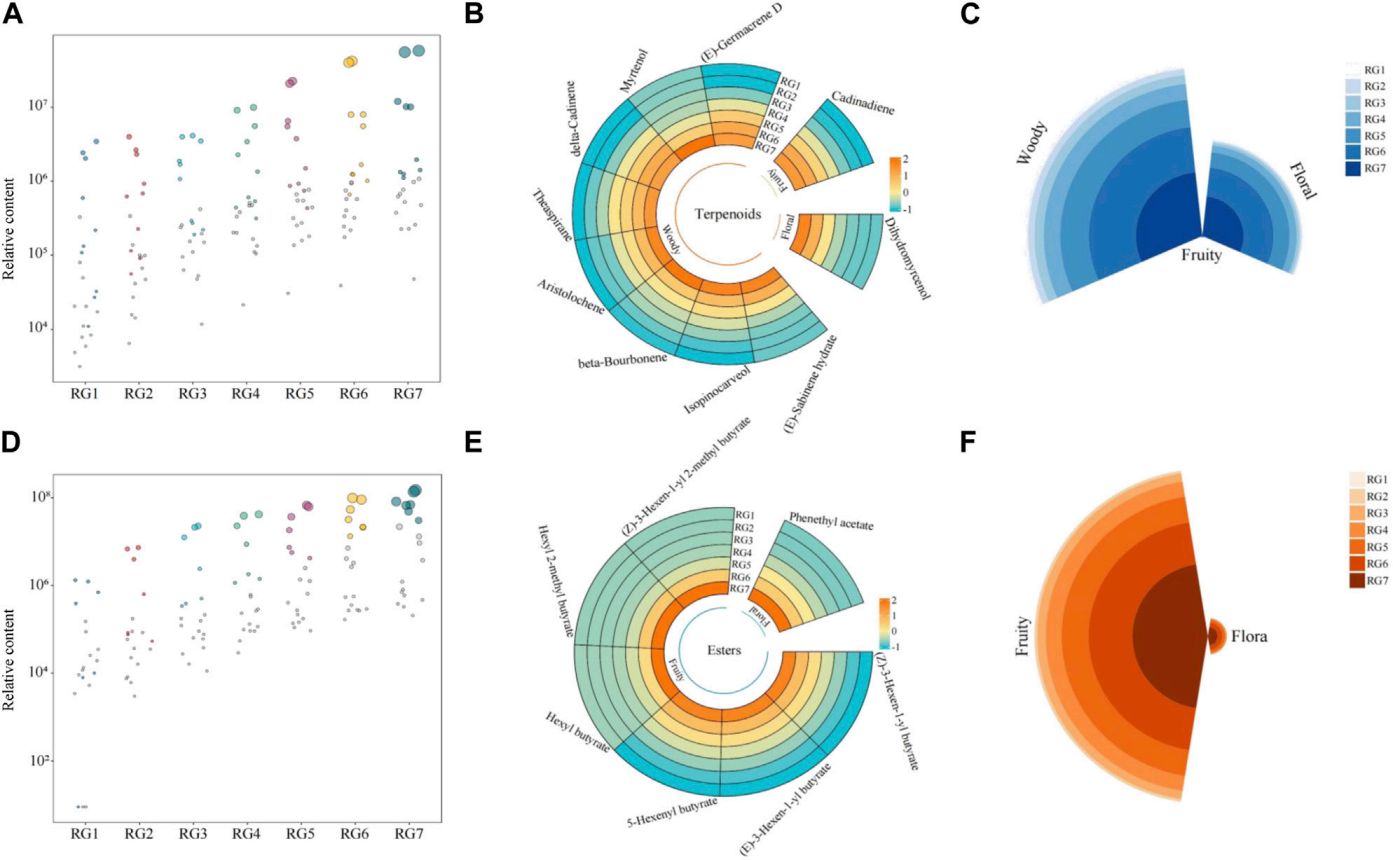
Screening of characteristic compounds and analysis of their odor characteristics. Note: RG1: Fresh leaf; RG2: Withered leaf; RG3: First fermentation; RG4: Second fermentation; RG5: Third fermentation; RG6: Fourth fermentation; RG7: Fifth fermentation; **(A)** Bubble characteristic map analysis of terpenoids; **(B)** Analysis of the content and odor characteristics of terpenoids; **(C)** Intensity analysis of odor characteristics of terpenoids; **(D)** Bubble characteristic map analysis of esters; **(E)** Analysis of the content and odor characteristics of esters; **(F)** Intensity analysis of odor characteristics of esters.

Based on the screening of key compounds, the bubble characteristic map was further used to screen the characteristic compounds in terpenoids and esters. The results of the bubble characteristic map analysis of terpenoids showed (Figure 5A) that a total of 10 characteristic terpenoids were obtained, which accounted for more than 90% of the key terpenoids in the content. Further analysis revealed that the odor characteristics of the 10 terpenoids were floral, fruity and woody, among which the odor characteristic of dihydromyrcenol was floral, the odor characteristic of cadinadiene was fruity, and the odor characteristic of the remaining 8 terpenoids was woody (Figure 5B). The results of the intensity analysis of odor characteristics showed (Figure 5C) that the odor characteristics presented by the characteristic terpenoids were predominantly woody. The results of the bubble characteristic map analysis of the esters showed (Figure 5D) that a total of 7 characteristic esters were obtained, which accounted for more than 90% of the key esters in the content. The 7 esters presented odor characteristics of fruit and floral, of which phenethyl acetate had floral odor characteristics, and the remaining 6 esters all had fruit odor characteristics (Figure 5E). The results of the intensity analysis of odor characteristics showed (Figure 5F) that the odor characteristics presented by the characteristic esters were mainly fruity.

**FIGURE 6:**
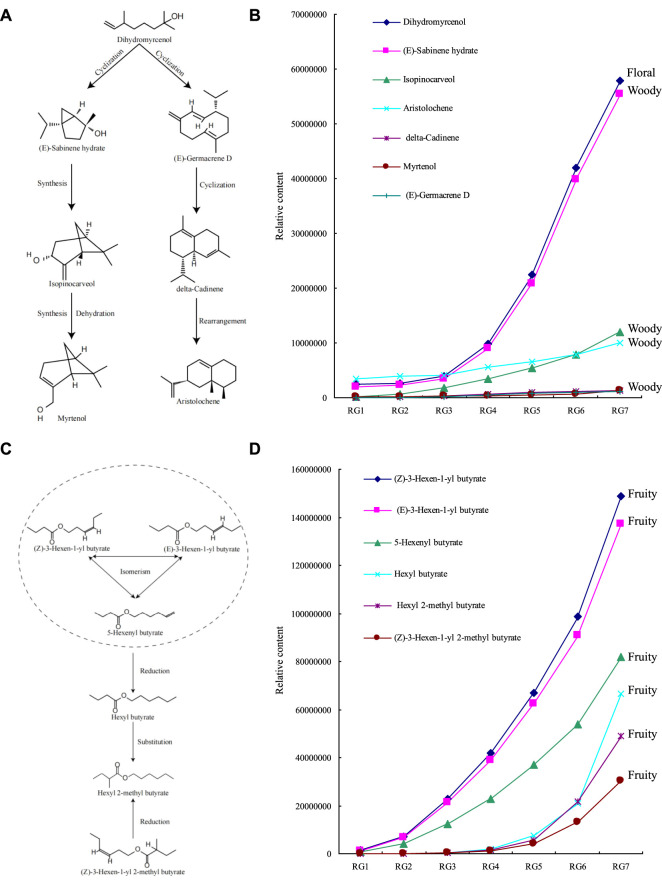
The contents of some characteristic volatile compounds and their transformation diagram. Note: RG1: Fresh leaf; RG2: Withered leaf; RG3: First fermentation; RG4: Second fermentation; RG5: Third fermentation; RG6: Fourth fermentation; RG7: Fifth fermentation; **(A)** Transformation diagram of characteristic terpenoids; **(B)** Content analysis of characteristic terpenoids; **(C)** Transformation diagram of characteristic esters; **(D)** Content analysis of characteristic esters.

On this basis, this study further analyzed the transformation relationships among the obtained characteristic compounds. The results of the transformation analysis of the characteristic terpenoids showed (Figure 6A) that dihydromyrcenol, as the basis of terpenoid transformaton, underwent a cyclization reaction and then rearranged to form (E)-sabinene hydrate, the three-membered ring of (E)-sabinene hydrate underwent a condensation reaction and further rearranged to form isopinocarveol, isopinocarveol underwent condensation to form Myrtenol; meanwhile, dihydromyrcenol could also undergo cyclization and synthesis reaction to form (E)-germacrene D, (E)-germacrene D further cyclized to form delta-cadinene, and delta-cadinene was isomerized with aristolochene. Further analysis revealed that the highest content of characteristic terpenoids was found in dihydromyrcenol, and it increased with the extension of the processing process (Figure 6B). Secondly, the main odor characteristic of dihydromyrcenol was floral, while the main odor characteristic of the other terpenoids was woody. It can be seen that the content of dihydromyrcenol increased continuously with the extension of the processing process, and the floral aroma of the tea became more intense; meanwhile, the increase of dihydromyrcenol content also provided an important basis for the subsequent synthesis of terpenoids with mainly woody aroma, especially (E)-sabinene hydrate.

The results of the transformation analysis of the characteristic esters (Figure 6C) indicated that (Z)-3-hexen-1-yl butyrate, (E)-3-hexen-1-yl butyrate, and 5-hexenyl butyrate were isomeric, especially (Z)-3-hexen-1-yl butyrate and (E)-3- hexen-1-yl butyrate were a pair of cis-trans isomers, and the C=C of all three could undergo hydrogenation and reduction to form hexyl butyrate, and hexyl butyrate continued to undergo substitution of α-H of carbonyl to form hexyl 2-methyl butyrate, while the C=C of (Z)-3-hexen-1-yl 2-methyl butyrate could undergo hydrogenation and reduction to form hexyl 2-methyl butyrate. Further analysis revealed that the odor characteristics of the characteristic esters all presented fruity, and their contents tended to increase with the extension of the processing process, and the top three compounds in the content were (Z)-3-hexen-1-yl butyrate, (E)-3-hexen-1-yl butyrate, and 5-hexenyl butyrate, which provided the basis for the synthesis of other key esters (Figure 6D). It can be seen that with the extension of the processing process, the content of (Z)-3-hexen-1-yl butyrate, (E)-3-hexen-1-yl butyrate, and 5-hexenyl butyrate increased continuously, and the fruity aroma of tea became more intense; meanwhile, the increase of the content of the three compounds also provided the basis for the subsequent synthesis of other eaters presenting fruit odor.

## 4 Conclusion

In this study, the effects of the first stage of preliminary processing of Wuyi rock tea on volatile compounds and their odor characteristics were analyzed. The results showed that the amount of volatile compounds did not change significantly during the first stage (fresh leaves picking, withering and fermentation), but the content changed significantly. Terpenoids, heterocyclic compounds, esters in tea leaves were mostly and significantly affected by these processing processes. The results of OPLS-DA model analysis showed that 41 key compounds (VIP >1) could distinguish between samples at different processing steps. Analysis of the bubble characteristic map showed that 17 characteristic compounds were obtained, including 10 terpenoids and 7 esters. The main odor characteristic of terpenoids were woody aroma, followed by floral aroma, and the formation of floral aroma was mainly from dihydromyrcenol and the formation of woody aroma was mainly from (E)-sabinene, isopinocarveol, aristolochene, delta-cadinene, myrtenol, (E)-germacrene D, etc, while, dihydromyrcenol was the basis of synthesis of these 6 terpenoids. The main odor characteristics presented by characteristic esters were fruity aromas. Fruity aroma formation was mainly derived from (Z)-3-hexen-1-yl butyrate, (E)-3-hexen-1-yl butyrate, 5-hexenyl butyrate, hexyl butyrate, hexyl 2-methyl butyrate, and (Z)-3-hexen-1-yl 2-methyl butyrate. Secondly, further analysis revealed that (Z)-3-hexen-1-yl butyrate, (E)-3-hexen-1-yl butyrate and 5-hexenyl butyrate belong to isomers and were the basis for the synthesis of other esters.

In conclusion, the first stage of preliminary processing of Wuyi rock tea mainly formed special floral, woody and fruity aromas of tea, and the formation of floral and woody aromas was mainly dominated by terpenoids, of which dihydromyrcenol was the key to the formation of floral aroma, and the other six characteristic terpenoids were the key to the formation of woody aroma. Synthesis of the above six terpenoids depended on the content of dihydromyrcenol. The formation of fruity aroma was mainly dominated by six ester compounds, and the key to fruity aroma intensity lay in the content of (Z) -3-hexen-1-yl butyrate, (E) -3-hexen-1-yl butyrate and 5-hexenyl butyrate. This study provided an important theoretical and practical basis for improving the preliminary processing of Wuyi rock tea.

## Data Availability

The datasets presented in this study can be found in online repositories. The names of the repository/repositories and accession number(s) can be found in the article/Supplementary Material.
