# Supplementary material for: Contribution of traditional deep fermentation to volatile metabolites and odor characteristics of Wuyi rock tea
Source: Front Bioeng Biotechnol. 2023 May 16;11:1193095. doi: 10.3389/fbioe.2023.1193095 (PMC10228688; doi:10.3389/fbioe.2023.1193095)
Supplement: Supplementary file 1 [file DataSheet1.PDF]

Supplementary Materials

Contribution of traditional deep fermentation to volatile metabolites and odor characteristics of Wuyi rock tea

Xiaoli Jia[1,†], Yuhua Wang[2,†], Qisong Li[1], Qi Zhang[1], Ying Zhang[1], Shaoxiong Lin[3], Pengyuan Cheng[3], Meihui Chen[1], Mengru Du[1], Jianghua Ye[1], Haibin Wang[1,*]

[1] College of Tea and Food, Wuyi University, Wuyishan 354300, China

[2] College of Life Science, Fujian Agriculture and Forestry University, Fuzhou 350002, China

[3] College of Life Science, Longyan University, Longyan 364012, China

* Correspondence: w13599084845@sina.com (H.W.)

† These authors contributed equally to this work.

Note :

RG1: Fresh leaf
RG2: Withered leaf
RG3: First fermentation
RG4: Second fermentation
RG5: Third fermentation
RG6: Fourth fermentation
RG7: Fifth fermentation

Table S1 Analysis of the quantity of volatile metabolites in tea leaves detected at different processing steps

Classification of compounds	Quantity	Proportion
Amine	15	1.99%
Alcohol	62	8.22%
Aromatics	50	6.63%
Phenol	18	2.39%
Nitrogen compounds	8	1.06%
Sulfur compounds	7	0.93%
Aldehyde	48	6.37%
Acid	20	2.65%
Terpenoids	152	20.16%
Hydrocarbons	65	8.62%
Ketone	65	8.62%
Heterocyclic compound	124	16.45%
Esters	120	15.92%
Total	754	

Table S2 Analysis of the relative content of volatile metabolites in tea leaves at different processing steps (10^9)

	RG1	RG2	RG3	RG4	RG5	RG6	RG7
Amine	0.155±0.021	0.141±0.012	0.145±0.004	0.175±0.019	0.155±0.013	0.152±0.020	0.169±0.028
Alcohol	0.170±0.060	0.225±0.039	0.282±0.039	0.349±0.038	0.411±0.049	0.464±0.046	0.622±0.079
Aromatics	0.108±0.008	0.114±0.017	0.172±0.025	0.297±0.012	0.445±0.062	0.611±0.049	0.751±0.073
Phenol	0.018±0.003	0.018±0.001	0.020±0.003	0.024±0.001	0.028±0.001	0.030±0.001	0.035±0.006
Nitrogen compounds	0.046±0.004	0.044±0.005	0.055±0.004	0.069±0.006	0.076±0.005	0.073±0.003	0.071±0.008
Sulfur compounds	0.015±0.000	0.015±0.001	0.021±0.004	0.033±0.002	0.045±0.003	0.050±0.003	0.050±0.004
Aldehyde	0.054±0.007	0.053±0.002	0.074±0.011	0.113±0.010	0.152±0.013	0.182±0.012	0.210±0.024
Acid	0.004±0.003	0.009±0.003	0.011±0.003	0.014±0.001	0.019±0.003	0.021±0.003	0.029±0.003
Terpenoids	0.599±0.039	0.558±0.041	0.838±0.132	1.358±0.055	1.927±0.188	2.511±0.079	2.811±0.243
Hydrocarbons	0.124±0.003	0.115±0.008	0.149±0.015	0.215±0.010	0.277±0.035	0.352±0.023	0.408±0.034
Ketone	0.146±0.018	0.130±0.007	0.197±0.028	0.300±0.035	0.389±0.029	0.464±0.028	0.548±0.077
Heterocyclic compound	0.295±0.019	0.316±0.035	0.550±0.209	1.031±0.005	1.267±0.116	1.475±0.074	1.611±0.096
Esters	0.163±0.047	0.250±0.059	0.450±0.148	0.794±0.043	1.280±0.154	1.878±0.227	2.611±0.294
Total	1.898±0.177	1.989±0.193	2.967±0.612	4.773±0.238	6.471±0.659	8.263±0.509	9.926±0.958

Table S3 Analysis of the proportion of volatile metabolites in tea leaves at different processing steps (%)

	RG1	RG2	RG3	RG4	RG5	RG6	RG7
Amine	8.16	7.07	4.89	3.66	2.39	1.84	1.70
Alcohol	8.98	11.32	9.51	7.32	6.35	5.61	6.26
Aromatics	5.69	5.75	5.81	6.22	6.88	7.39	7.57
Phenol	0.95	0.92	0.69	0.50	0.43	0.36	0.35
Nitrogen compounds	2.45	2.22	1.86	1.45	1.17	0.88	0.71
Sulfur compounds	0.79	0.74	0.72	0.69	0.70	0.61	0.50
Aldehyde	2.85	2.67	2.51	2.37	2.35	2.21	2.11
Acid	0.22	0.43	0.38	0.30	0.29	0.26	0.30
Terpenoids	31.57	28.07	28.25	28.45	29.78	30.38	28.32
Hydrocarbons	6.53	5.78	5.03	4.50	4.29	4.26	4.11
Ketone	7.68	6.55	6.66	6.29	6.01	5.61	5.52
Heterocyclic compound	15.54	15.88	18.54	21.59	19.57	17.85	16.23
Esters	8.60	12.59	15.16	16.64	19.78	22.73	26.31

Table S4 Quantity analysis of volatile metabolites with variation in tea leaves at different processing steps

	RG2 vs RG1	RG3 vs RG2	RG4 vs RG3	RG5 vs RG4	RG6 vs RG5	RG7 vs RG6
Up-regulated	389	533	697	519	492	603
Down-regulated	249	192	56	235	261	150
No-significant	116	29	1	0	1	1

Table S5 Classification and quantity analysis of 179 volatile compounds

**Classification of compounds**	**Quantity**	**Proportion**
Amine	2	1.12%
Alcohol	13	7.26%
Aromatics	10	5.59%
Phenol	4	2.23%
Nitrogen compounds	2	1.12%
Aldehyde	8	4.47%
Acid	8	4.47%
Terpenoids	45	25.14%
Hydrocarbons	9	5.03%
Ketone	16	8.94%
Heterocyclic compound	31	17.32%
Esters	31	17.32%
Total	179	

Table S6 Analysis of the relative content of 179 volatile compounds (10^9)

	RG1	RG2	RG3	RG4	RG5	RG6	RG7
Amine	0.0002±0.0000	0.0002±0.0000	0.0002±0.0000	0.0005±0.0000	0.0010±0.0002	0.0018±0.0003	0.0024±0.0002
Alcohol	0.0012±0.0004	0.0037±0.0019	0.0137±0.0008	0.0341±0.0031	0.0745±0.0079	0.1482±0.0291	0.2685±0.0431
Aromatics	0.0019±0.0002	0.0046±0.0024	0.0163±0.0009	0.0631±0.0009	0.1477±0.0313	0.2753±0.0350	0.4307±0.0497
Phenol	0.0007±0.0001	0.0012±0.0001	0.0014±0.0003	0.0019±0.0000	0.0023±0.0004	0.0029±0.0003	0.0038±0.0008
Nitrogen compounds	0.0001±0.0000	0.0001±0.0000	0.0001±0.0000	0.0002±0.0000	0.0004±0.0001	0.0005±0.0001	0.0016±0.0005
Aldehyde	0.0060±0.0045	0.0067±0.0020	0.0101±0.0027	0.0158±0.0018	0.0230±0.0035	0.0303±0.0043	0.0426±0.0050
Acid	0.0005±0.0001	0.0009±0.0004	0.0024±0.0011	0.0051±0.0003	0.0093±0.0014	0.0147±0.0022	0.0201±0.0023
Terpenoids	0.0104±0.0017	0.0257±0.0031	0.0991±0.0056	0.3064±0.0249	0.6124±0.0729	1.0023±0.0335	1.2279±0.0077
Hydrocarbons	0.0017±0.0004	0.0028±0.0010	0.0081±0.0018	0.0185±0.0006	0.0374±0.0065	0.0661±0.0079	0.0938±0.0038
Ketone	0.0021±0.0003	0.0065±0.0033	0.0204±0.0015	0.0485±0.0010	0.0912±0.0127	0.1440±0.0128	0.1950±0.0191
Heterocyclic compound	0.0097±0.0016	0.0127±0.0032	0.0258±0.0061	0.0592±0.0022	0.1210±0.0232	0.2129±0.0276	0.3151±0.0208
Esters	0.0053±0.0006	0.0513±0.0030	0.2090±0.0180	0.4676±0.0086	0.8628±0.0974	1.3607±0.1535	1.9575±0.2002
Total	0.0398±0.0037	0.1164±0.0588	0.4066±0.1137	1.0208±0.0081	1.9831±0.2549	3.2596±0.2945	4.5590±0.4131
